# High Coulomb Efficiency Sn–Co Alloy/rGO Composite Anode Material for Li–ion Battery with Long Cycle–Life

**DOI:** 10.3390/molecules28093923

**Published:** 2023-05-06

**Authors:** Ding Shen, Mengyuan Jia, Mingyue Li, Xiaofan Fu, Yaohan Liu, Wei Dong, Shaobin Yang

**Affiliations:** 1 College of Material Science and Engineering, Liaoning Technical University, Fuxin 123000, China; shending028@163.com (D.S.);; 2 Institute of Engineering Technology and Natural Science, Belgorod State University, Belgorod 308015, Russia

**Keywords:** lithium–ion battery, Sn–Co alloy, reduction of graphene oxide, coulomb efficiency

## Abstract

The low cycle performance and low Coulomb efficiency of tin-based materials confine their large–scale commercial application for lithium–ion batteries. To overcome the shortage of volume expansion of pristine tin, Sn–Co alloy/rGO composites have been successfully synthesized by chemical reduction and sintering methods. The effects of sintering temperature on the composition, structure and electrochemical properties of Sn–Co alloy/rGO composites were investigated by experimental study and first-principles calculation. The results show that Sn–Co alloys are composed of a large number of CoSn and trace CoSn_2_ intermetallics, which are uniformly anchored on graphene nanosheets. The sintering treatment effectively improves the electrochemical performance, especially for the first Coulomb efficiency. The first charge capacity of Sn–Co alloy/rGO composites sintered at 450 °C is 675 mAh·g^−1^, and the corresponding Coulomb efficiency reaches 80.4%. This strategy provides a convenient approach to synthesizing tin-based materials for high-performance lithium–ion batteries.

## 1. Introduction

Lithium–ion battery has attracted much attention in the field of portable electronic devices and electric vehicles because of their excellent characteristics, such as high energy density, high working voltage and long cycle life. However, the theoretical specific capacity of the most commonly used commercial graphite anode electrode is only mAh·g^−1^, which obviously limits the improvement of lithium storage capacity of lithium–ion batteries [1]. Therefore, exploring a new generation of anode materials with high capacity has become one of the important research fields of lithium–ion batteries. Metal tin has a high theoretical capacity (990 mAh·g^−1^, Li_4.4_Sn) [2], which is one of the most likely candidates for anode materials. However, the lithium storage process of metallic tin is complex and accompanied by a huge volume change (up to 300%), which leads to serious structural damage to metal tin and the continuous formation of solid electrolyte thin films (SEI) on the surface of newly broken tin particles [3,4]. These processes will aggravate the lower Coulomb efficiency and worse cycle performance of tin [5,6].

In order to solve the volume expansion of tin, one of the strategies is to use various forms of carbon materials with high electrical conductivity as carriers to synthesize nanocomposites containing Sn and carbon, such as activated carbon [7], carbon nanotubes [8,9], carbon fibers [10,11] and graphene [12,13]. In particular, graphene with high mechanical strength properties is used as a carrier to construct a volumetric expansion buffer structure, which can significantly improve the electrochemical performance of the composite electrode. In addition, the strategy of introducing O into metals to synthesize complex metal oxides can also significantly improve the structural stability and electronic properties of metal oxide electrode materials [14,15].

In the existing literature, the research is mainly focused on the use of one–dimensional graphene tubes, two–dimensional graphene sheets and three–dimensional graphene network structures to construct Sn/graphene composites. Mo et al. [16] synthesized a kind of tin nanoparticles/double–graphene–tube composite (Sn/DGT) by chemical deposition and heat treatment. The tin nanoparticles are confined in the one–dimensional double–graphene tubes, which minimizes the structural damage of tin nanoparticles. As a result, the first reversible capacity and Coulomb efficiency of Sn/DGT composites are 913 mAh·g^−1^ and 71.1%, respectively, at 0.2 A/g, and the capacity of Sn/DGT composites reached 918 mAh·g^−1^ after 500 cycles. Chen et al. [17] prepared a kind of tin–graphene nanocomposites (Sn–GNS) by microwave hydrothermal synthesis and hydrogen reduction method. Sn nanoparticles with 10~20 nm are sandwiched between two–dimensional graphene nanosheets with high conductivity and flexibility. The first reversible capacity and Coulomb efficiency of the Sn–GNS composite are 1407 mAh·g^−1^ and 65.9%, respectively, and the capacity after 30 cycles is about 899 mAh·g^−1^. Qin et al. [18] successfully synthesized a 3D Sn@G–PGNWs material composed of tin nanoparticles (5~30 nm) coated with a three-dimensional graphene shell by chemical vapor deposition. The interconnected three-dimensional porous graphene network formed by a good elastic graphene shell buffers the volume expansion and improves the integrity of the overall structure of the electrode. The first reversible capacity and Coulomb efficiency of the 3D Sn@G–PGNWs composite at 0.2 A·g^−1^ is about 1245 mAh·g^−1^ and 69%, respectively. Meanwhile, the capacity of the 3D Sn@G–PGNWs composite remains 682 mAh·g^−1^ after 1000 cycles at 0.2 A·g^−1^, showing good rate performance and cycle performance.

From the above, the morphology and structure of graphene have a significant impact on the electrochemical performance of nanocomposites, in which the three-dimensional interconnected graphene network structure has a significant contribution to the improvement of structural stability of the composites. At the same time, N, S doping can further improve the lithium storage properties of the composites [19,20,21]. Liu et al. [22] synthesized a kind of Sn@N-doped graphene electrode material (Sn@NG) by high temperature pyrolysis using cyanamide as N source, in which ultrafine tin nanoparticles (2~3 nm) are uniformly embedded in N–doped graphene (NG) network. The wrinkled NG provides good electrical conductivity, rich defects, high specific surface area and large mesopore volume. The first discharge capacity and Coulomb efficiency of the Sn@NG material at 1 h·g^−1^ are 1054 mAh·g^−1^ and 52.1%. In addition, the capacity of Sn@NG material remained at 568 mAh·g^−1^ after 1000 cycles, showing good cycle stability.

Furthermore, the addition of alloy elements into the metal to form a second phase can significantly improve the structural stability and electronic properties of the materials [23,24]. For Sn-based alloys, the introduction of inactive metal *M* (*M* = Cu, Co, Fe, Ni, etc.) into the tin metal to form an Sn–M alloy can further buffer the volume change of metal tin. One of the earliest Sn–based anode materials for lithium–ion battery is Sn–Cu alloy. According to the binary metal phase diagram, Sn–Cu alloy is mainly composed of single phases such as pure tin, Cu_6_Sn_5_ and Cu_3_Sn intermetallics or mixed substances [25]. Zhu et al. [26] prepared a kind of Sn–Cu–graphene composite (Sn–Cu–GNS) by chemical reduction method, in which Sn and amorphous Cu nanoparticles are uniformly dispersed on graphene. Due to the synergistic action of tin, copper and graphene, the volume change of the Sn–Cu–GNS material is inhibited, and the electron transfer is promoted, thus obtaining excellent electrochemical performance. The first reversible capacity and Coulomb efficiency of the Sn–Cu–GNS material are about 525 mAh·g^−1^ and 63.6% at 500 mA·g^−1^, and the capacity can reach 643 mAh·g^−1^ after 100 cycles. Wang et al. [27] prepared an electrode material (SnCo/NC) composed of Sn–Co alloy and N–doped graphene by freeze-drying and heat treatment, in which CoSn and CoSn_2_ nanoparticles are embedded in the carbon skeleton of nitrogen–doped graphene. The first reversible capacity and Coulomb efficiency of the SnCo/NC material are 1017 mAh·g^−1^ and 63.8% at 0.2 A/g, and the capacity of the composite can still reach 810 mAh·g^−1^ after 600 cycles at 1 A·g^−1^, showing excellent lithium storage performance. Xin et al. [28] prepared a kind of Fe_0.74_Sn_5_@RGO composite by chemical reduction method. Corresponding to the FeSn_5_ crystal structure, the composite structure composed of defective Fe_0.74_Sn_5_ nanoparticles dispersed on graphene adapts to the change of volume structure and shortens the transport distance of Li ions and electrons. The first reversible capacity and Coulomb efficiency of Fe_0.74_Sn_5_@RGO composite are 957 mAh·g^−1^ and 62.9%, keeping 674 mAh·g^−1^ after 100 cycles. In addition, the introduction of polymers into alloys or metal oxides can also improve the structural stability of electrode materials, which has become a new research field of polymer-based composites promising for practical applications [29,30].

Based on the above literature, the formation of nanocomposite structure and the synthesis of Sn–M alloy are the optimal strategies to improve the electrochemical performance of tin–based materials. However, it is found that the common disadvantage of these tin–containing anode materials is that the first Coulomb efficiency is low, which can not meet the requirements of the new generation of lithium–ion batteries. It is urgent and important to synthesize tin–based materials with long cycle life and high Coulomb efficiency.

Here, Sn–Co alloy/rGO composites have been successfully prepared by chemical reduction and then sintering treatment using graphene oxide as a carrier, in which Sn–Co nano–alloys are uniformly anchored on graphene. This structure has a variety of functions and advantages: (1) The synthesized nano–sized Sn–Co alloy has a higher resistance to structural destruction because of its small particle size. (2) The introduction of Co atoms in Sn–Co alloy as an inert medium helps to buffer the volume expansion of metal Sn. (3) The buffering effect formed by the good mechanical properties of graphene can further improve the structural stability of Sn–Co alloy, and the good electronic conductivity of the electrode is ensured because graphene has good electrical conductivity. (4) Sintering treatment can increase the grains and particles of Sn–Co alloy, thus improving the first Coulomb efficiency of Sn–Co alloy/rGO composites. The results show that Sn–Co alloy/rGO composites have good cycle performance as anode materials for lithium–ion batteries, especially with high first Coulomb efficiency.

## 2. Results and Discussions

### 2.1. Microstructure and Composition

**Figure 1:**
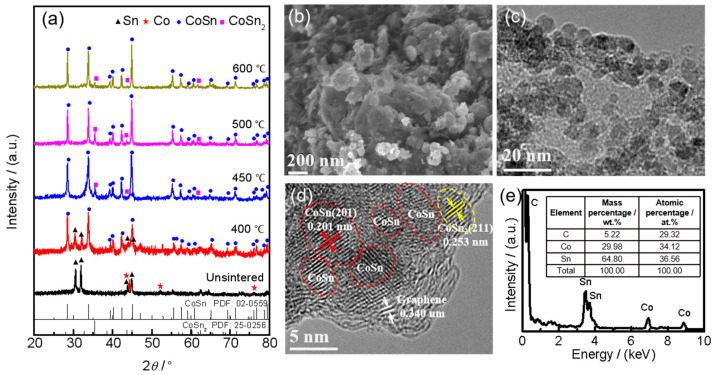
The XRD patterns of the synthesized Sn–Co alloy/rGO composite (**a**), SEM image (**b**), TEM image (**c**), HRTEM image (**d**) and EDS (**e**) of Sn–Co alloy/rGO composite sintered at 500 °C.

The microstructure analysis of the synthesized Sn–Co alloy/rGO composite is shown in Figure 1. As can be seen from Figure 1a, the Sn–Co alloy/rGO composite without sintered is composed of pure Sn and Co metal according to the standard data of X-ray diffraction. After sintering at 400 °C, the phase of Sn–Co alloy/rGO composite transformed into CoSn intermetallics, while a small amount of unreacted pure Sn metal remained. After sintering at 450 °C, a small amount of CoSn_2_ intermetallics were newly formed in the Sn–Co alloy/rGO composite. With the increase of sintering temperature, the phase composition of Sn–Co alloy/rGO composite no longer changes, but the FWHM of the XRD spectrum of all phases of the composite decreases, indicating that the grains of CoSn and CoSn_2_ intermetallics continue to increase or the degree of crystallization increases. Similar phase compositions have also been reported in Sn–Co alloy; for example, CoSn and CoSn_2_ intermetallics coexist in Sn–Co alloy prepared by mechanical ball milling [31]. However, there is no obvious carbon diffraction peak in the XRD spectrum in Figure 1a, which may be due to an amorphous structure of graphene.

The typical SEM and TEM images in Figure 1b,c show the surface morphologies of Sn–Co alloy/rGO composite after sintering at 500 °C, and the morphologies of other samples are shown in Figure S1. It can be seen that the Sn–Co alloy/rGO composite is nearly spherical particles with a diameter range of about 10 to 100 nm. From the HRTEM in Figure 1d, it is found that the main regions of Sn–Co alloy/rGO composites are (201) planes of CoSn intermetallics with a plane distance of 0.201 nm, while a very small part of the regions are (211) planes of CoSn_2_ intermetallics with the plane distance of 0.253 nm. Figure 1e shows the energy dispersive X-ray spectroscopy (EDS) results of Sn–Co alloy/rGO composite sintered at 500 °C, in which the atomic ratio of Sn to Co is close to 1:1. This is basically consistent with the raw material ratio of material synthesis and the results of XRD analysis.

**Figure 2:**
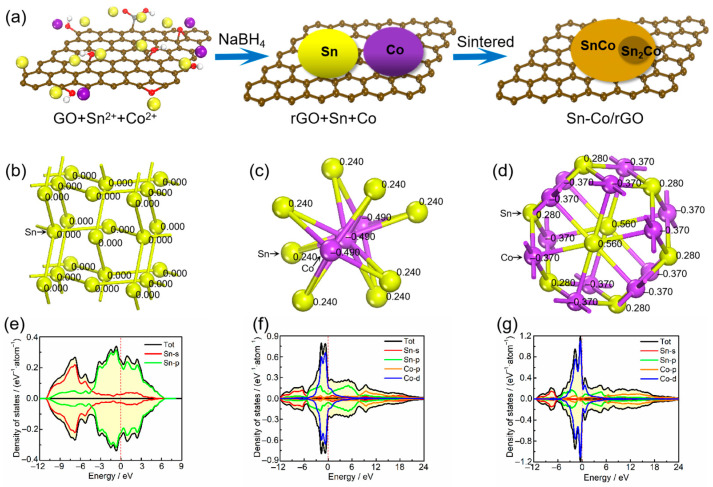
The schematic sketch of Sn–Co alloy/rGO composite (**a**), the crystal structure (atomic population) (**b**) and density of states (**e**) for pure Sn, the crystal structure (atomic population) (**c**) and density of states (**f**) for CoSn_2_ intermetallics, the crystal structure (atomic population) (**d**) and density of states (**g**) for CoSn intermetallics.

During the synthesis of Sn–Co alloy/rGO composite, Sn^2+^ and Co^2+^ in the solution are electrostatically adsorbed on graphene oxide near the oxygen-containing functional groups, such as the hydroxyl group, carboxyl group and epoxy group [32,33]. Then, these metal ions and graphene oxide are reduced to metal Sn, Co and graphene, respectively, by NaBH_4_ reduction. In the subsequent sintering process, Sn and Co atoms diffuse on graphene to form an Sn–Co alloy formed by a large number of CoSn intermetallics and a small amount of CoSn_2_ intermetallics. The schematic sketch of Sn–Co alloy/rGO composite is shown in Figure 2a.

In order to further analyze the electronic structures of pure Sn metal, CoSn_2_ and CoSn intermetallic in Sn–Co alloy/rGO composite from the atomic level, the electronic densities of states and atomic population of the three materials were calculated by first principles, the results are shown in Figure 2b–d. As can be seen from Figure 2b–d, there is no charge transfer between Sn atoms in pure tin, and all Sn atoms share a charge. For CoSn_2_ intermetallics, each Sn atom transfers 0.24 e to the Co atom on average. Similarly, for CoSn intermetallics, the average charge transfer from each Sn atom to the Co atom is 0.37 e. It can be inferred that the Sn–Co bond in CoSn_2_ and CoSn intermetallics is a metal bond with certain ionic characteristics, according to the difference in electronegativity between Sn and Co elements [34]. It can be seen from the Figure 2e–g that the density of states of pure tin near the Fermi level is mainly contributed by the s orbitals and p orbitals of Sn atoms, while the density of states of CoSn_2_ and CoSn intermetallics is mainly contributed by the s and p orbitals of Sn atoms and the p orbitals and d orbitals of Co atoms, and the contribution of Co atoms is more. Therefore, pure tin, CoSn_2_ and CoSn intermetallics have higher densities of states near the Fermi level, and the densities of states increase from 0.25 to 0.37 with the increase of Co content. The results show that all of them have good electrical conductivity, and the electrical conductivity of Sn–Co alloy increases with the increase of Co content.

### 2.2. Electrochemical Performance

**Figure 3:**
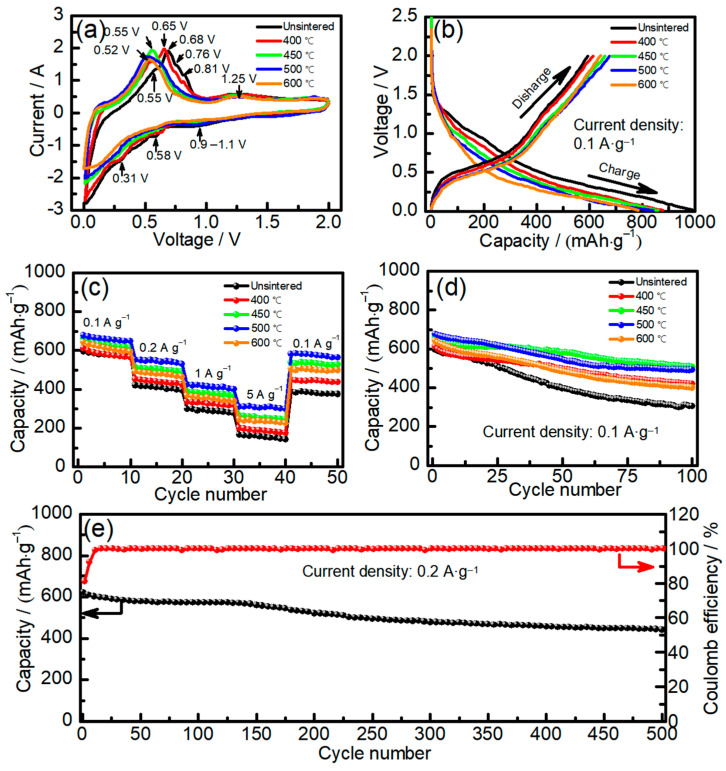
The cyclic voltammetry curves of Sn–Co alloy/rGO composite (**a**), the constant current charge–discharge curves of Sn–Co alloy/rGO composite (**b**), the cycle performance curves of Sn–Co alloy/rGO composite (**c**), the rate performance curves of Sn–Co alloy/rGO composite (**d**), the long cycle test of Sn–Co alloy/rGO composite sintered at 450 °C (**e**).

Figure 3 shows the electrochemical performance of Sn–Co alloy/rGO composites. In order to analyze the lithium intercalation mechanism of electrode materials during charge and discharge, Sn–Co alloy/rGO composites were tested by cyclic voltammetry and the results are shown in Figure 3a. It can be seen from Figure 3a that there are obvious reduction peaks at 0.9~1.1 V and below 0.8 V in the Sn–Co alloy/rGO composites without sintered, which is similar to the CV curve of pure tin [35]. The peak at 0.9~1.1 V is usually attributed to some irreversible reactions of forming SEI interface on the surface of active material particles [36]. The peak below 0.8 V corresponds to the process that pure tin reacts with lithium to form Li*_x_*Sn alloy (Sn + *x*Li^+^ + *x*e → Li*_x_*Sn, 0 ≤ *x* ≤ 4.4) [37]. In the process of reverse scanning, some obvious oxidation peaks were observed at 0.55 V, 0.68 V, 0.76 V and 0.81 V, respectively. The corresponding Li*_x_*Sn alloys were dealloyed to form Li_3.5_Sn, LiSn, Li_2_Sn_5_ and pure Sn metal [38]. After sintering, the oxidation peak of Sn–Co alloy/rGO composites shifts to the left, and a wide oxidation peak appears in the range of 0.52~0.65 V, which is mainly due to the formation of Sn–Co alloy during sintering. This is similar to the results confirmed by Zheng et al. [39] in the oxidation peak of Sn–Co alloy appears at 0.5~0.6 V.

Figure 3b shows the first charge–discharge curve of Sn–Co alloy/rGO composite at 100 mAh g^−1^. It can be seen from Figure 3b that the Sn–Co alloy/rGO composite shows a weak platform at about 1.1 V and a tilted platform below 0.80 V, which correspond to the reduction peaks in the CV curve in Figure 3a. It can also be obtained from Figure 2b that the first charge capacity and discharge capacity of Sn–Co alloy/rGO composite without sintered are 995 and 595 mAh·g^−1^, respectively, and the corresponding first Coulomb efficiency is 59.8%. After sintering, the first charge capacity of Sn–Co alloy/rGO composite decreases gradually, while the first discharge capacity increases at first and then decreases. When sintering at 500 °C, the first charge capacity of Sn–Co alloy/rGO composite is 840 mAh·g^−1^, the first discharge capacity reaches the maximum, which is 675 mAh·g^−1^, and the corresponding first Coulomb efficiency reaches 80.4%. This may be due to the increase in the grain size of Sn–Co alloy in Sn–Co alloy/rGO composites, which leads to the increase in Coulomb efficiency. It is well known that nanomaterials have the disadvantage of low Coulomb efficiency [40], so increasing grain size and particle size is an effective strategy to improve Coulomb efficiency.

Figure 3c shows the rate performance of Sn–Co alloy/rGO composite with different sintering temperatures. It is found that the discharge capacity of Sn–Co alloy/rGO composite sintered at 500 °C is 675, 552, 425 and 311 mAh·g^−1^ at 100, 200, 1000 and 5000 mA·g^−1^, respectively. When the current density returns to 100 mA·g^−1^, the discharge capacity of Sn–Co alloy/rGO composite reaches 580 mAh·g^−1^, which shows a good rate performance.

Figure 3d shows the cycle performance of Sn–Co alloy/rGO composites with different sintering temperatures at 100 mA·g^−1^. The discharge capacity of Sn–Co alloy/rGO composites without sintered is only 303 mAh·g^−1^ after 100 cycles. After sintering, the cycle properties of Sn–Co alloy/rGO composites increase at first and then decrease with the increase of sintering temperature. The cycle performance of Sn–Co alloy/rGO composites sintered at 450 °C reached the maximum, and the discharge capacity is 508 mAh·g^−1^ after 100 cycles. The long cycle test of Sn–Co alloy/rGO composite sintered at 450 °C was carried out at 200 mA·g^−1^, and the results are shown in Figure 2e. It can be seen that the capacity of Sn–Co alloy/rGO composite reduces to 443 mAh·g^−1^ after 500 cycles from 622 mAh·g^−1^, and the capacity retention rate is 71.2%. Therefore, the Sn–Co alloy/rGO composites prepared by chemical reduction and then sintering treatment shows good cycle performance, especially the first cycle Coulombic efficiency is high compared with the literature, as shown in Table S2.

**Figure 4:**
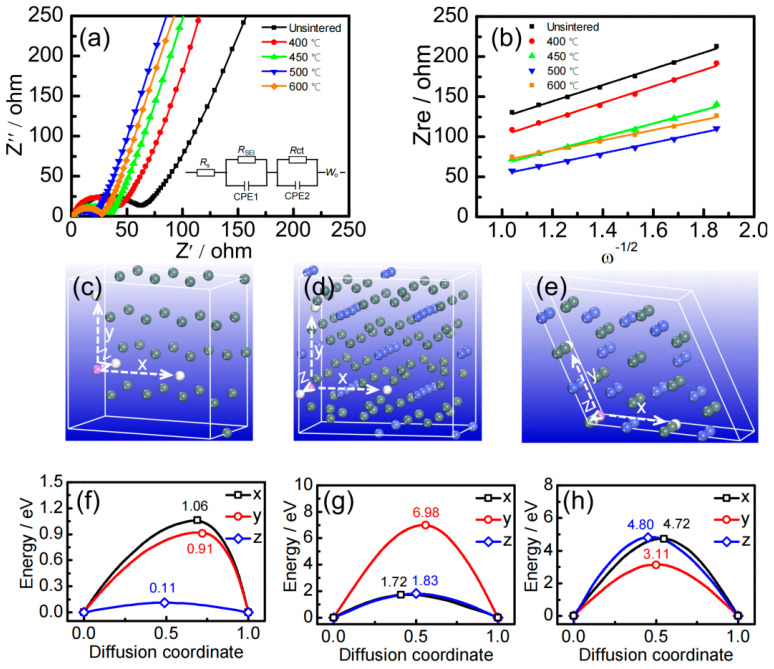
The AC impedance spectra (**a**) and fitting curve (**b**) of Sn–Co alloy/rGO composites, the diffusion direction (**c**) and diffusion energy barrier (**f**) for pure Sn, the diffusion direction (**d**) and diffusion energy barrier (**g**) for CoSn_2_ intermetallics, the diffusion direction (**e**) and diffusion energy barrier (**h**) for CoSn intermetallics.

In order to investigate the interface properties of electrode materials, the AC impedance spectra of Sn–Co alloy/rGO composites with different sintering temperatures were measured and the results are shown in Figure 4a. The internal resistance *R*_s_, the impedance of lithium–ion diffusion in SEI *R*_SEI_ and the charge transfer impedance between active material and electrolyte *R*_ct_ obtained by fitting equivalent circuit model [41] are recorded in Table 1.

The diffusion coefficient of lithium–ion can be calculated by formula [42]:

(1) $$D_{{\mathrm{Li}}^{+}}=0.5{\left[\frac{V_{m}}{FS\sigma }\left(-\frac{dE}{dx}\right)\right]}^{2}$$

Here, *V*_m_ is the molar volume (cm^3^·mol^−1^), *F* is the Faraday constant (9.6485 × 10^4^ C·mol^−1^), *S* is the electrode surface area (cm^2^), *σ* is the Warburg coefficient, which is the slope of the fitting line in Figure 4b, and *dE*/*dx* is the slope of the Coulomb titration line.

From the data in Table 1, it can be seen that with the increase of sintering temperature, the *R*_S_ and *R*_SEI_ of Sn–Co alloy/rGO composites decrease gradually, and *R*_ct_ decreases at first and then increases, which is mainly due to the formation of Sn_2_Co intermetallics. However, when the sintering temperature exceeds 500 °C, the grains are easy to grow, which is not conducive to the diffusion of lithium ions in the solid phase, and finally leads to the decrease of the diffusion coefficient.

In order to clarify the lithium–ion diffusion of pure Sn, CoSn_2_ and CoSn intermetallics in Sn–Co alloy/rGO composites on an atomic scale, the diffusion energy barriers of lithium atoms in these phases were calculated by first principles. The diffusion direction and diffusion energy barrier are shown in Figure 4c–f.

It can be seen from Figure 4c that the diffusion energy barriers of lithium ions of pure tin, CoSn_2_ and CoSn intermetallics are anisotropic in different directions. For example, the diffusion energy barrier of lithium–ion along the Z-axis in pure tin metal is the lowest compared with the X- and Y-axis, only 0.11 eV, which is close to the energy barrier of Sn (0.04 eV) in the literature [43]. The diffusion energy barriers of lithium ions in CoSn_2_ intermetallics along the X-axis and *Z*-axis are relatively lower, which is 1.72 eV and 1.83 eV, respectively. For CoSn intermetallics, the diffusion energy barrier of lithium ions along the Y-axis is the lowest, but the value is as high as 3.11 eV. It can be concluded that the diffusion energy barriers of lithium–ion increases gradually in the following order: pure Sn < CoSn_2_ < CoSn. Here, the addition of cobalt to tin can effectively improve the cycle performance, but excess cobalt will significantly hinder the dynamic diffusion of lithium atoms in Sn–Co alloy.

## 3. Materials and Methods

### 3.1. Preparation of Materials

Synthesis of rGO. Graphene oxide (GO) was prepared by an improved Hummers method [44]. Firstly, 5 g NaNO_3_ and 230 mL concentrated H_2_SO_4_ were added to the flask with 500 mL in an ice water bath. When the solution temperature dropped to 0 °C, 10 g graphite was added to the flask and stirred for 15 min. Then, 40 g KMnO_4_ was added to the flask within 30 min and stirred at 10~15 °C for 90 min. The solution was heated to 35~40 °C and stirred for 30 min. Third, 700 mL of deionized water was added to the flask within 30 min. The temperature of the solution in the flask was kept between 90 °C and 95 °C by controlling the rate of adding water. Then, the H_2_O_2_ with a mass fraction of 5 wt.% was added to the flask until no bubbles appeared and filtered while it was hot. Finally, the filtered cake was dissolved in 5 wt.% HCl solution, stir evenly and filter and repeat 3~4 times, and then wash to neutral with deionized water to obtain the required GO.

Synthesis of Sn–Co alloy/rGO composites. Firstly, Sn–Co alloy/rGO composite precursors were prepared by chemical reduction. The details were as follows: 22.6 g stannous chloride (SnCl_2_·2H_2_O) and 23.8 g CoCl_2_·6H_2_O were fully dissolved in 200 mL deionized water, then 1 g GO was added to the solution, ultrasonic for 2 h, and then a certain amount of sodium citrate and polyvinylpyrrolidone (PVP) was added to the solution, and then dispersed uniformly by ultrasonic 30 min. The resulting solution was labeled as A solution. Subsequently, 0.15 g NaOH was dissolved in 50 mL deionized water, then 1 g NaBH_4_ as a reducing agent was added, and the obtained solution was labeled as B solution. In an ice water bath, the B solution was slowly added to the A solution with stirring and continued stirring for 2 h, then filtered and washed with water until neutral pH, and the obtained powder was dried at 60 °C for 24 h in vacuum. Finally, the drying product was sintered at 400~600 °C for 2 h in a tube furnace protected by argon, and the target product Sn–Co alloy/rGO composites were prepared.

### 3.2. Materials Characterization

The crystal structure of the composite was characterized by X-ray diffraction (XRD, Shimadzu XRD–6100, Cu K*α* radiation, *λ* = 0.1542 nm). The surface morphology and microstructure of the materials were observed by scanning electron microscope (SEM, JEOL JSM–7500F) and transmission electron microscope (TEM, JEOL JEM–2010) with an energy dispersive X-ray spectrometer (EDS).

### 3.3. Electrochemical Measurements

The working electrode was prepared with the active material, conductive agent (acetylene black) and polyvinylidene fluoride (PVDF) according to the mass ratio of 85:5:10, and the active material in working electrode is ~3.0 mg/cm^2^. The 2032 button battery for lithium storage performance test was assembled by using metal Li sheet as the counter electrode with 1.0 mg, Celgard 2400 polypropylene membrane as the separator and 1.0 mol/L LiPF_6_/EC+DMC+DEC (Volume ratio 1:1:1) of 0.04 mL as the electrolyte.

The galvanostatic discharge–charge (GCD) was performed on a battery test system (Sunway, BTS–5 V 10 mA) with the voltage of 0.01~2.00 V (vs. Li^+^/Li). The three button batteries were charged and discharged 100 times, respectively, and the capacity retention rate between the maximum value and the minimum value was taken as the test result of long cycle performance. In the process of long cycle test, three button batteries were tested for 100 cycles, and the capacity retention between the maximum and the minimum was taken as the test results of the long cycle performance. The cyclic voltammetry (CV) curve was recorded using an electrochemical workstation (Chenhua, CHI604E) at a scan rate of 20 mV·s^−1^ in the voltage of 0.01~2.00 V. The electrochemical impedance spectroscopy (EIS) measurements were also carried out on the CHI604E electrochemical workstation with frequencies ranging from 100 kHz to 10 mHz.

### 3.4. Theoretical Calculation

According to the phase composition of Sn–Co alloy/rGO composite, the density charge and density of states of pure Sn metal, CoSn_2_ and CoSn intermetallics with lowest energy configuration were calculated using the CASTEP software package [45] of plane wave pseudopotential method based on density functional theory with the consideration of spin-polarized effect. The generalized gradient approximation (GGA) of Perdew–Burke–Ernzerh [46] of (PBE) approaches were employed for all the calculations. The electronic wave functions were expanded in a plane–wave basis set using a kinetic energy cutoff of 500 eV, and the interactions between ionic cores and valence electrons are described by ultrasoft pseudopotentials [47]. For pure Sn metal, CoSn_2_ and CoSn intermetallics, the K-point mesh [48] of 8 × 8 × 16, 8 × 8 × 7 and 11 × 11 × 12 was chosen for optimizing geometric configuration and analyzing the electronic properties. The transition states (TS) and barriers of the supercell (2 × 2 × 2) of pure Sn metal, Sn_2_Co and SnCo intermetallics were calculated using nudged elastic band method (NEB) [49].

## 4. Conclusions

Here, Sn–Co alloy/rGO composites have been successfully prepared by chemical reduction and then sintering using graphene oxide as a carrier. The metallic elemental tin and cobalt obtained by chemical reduction are diffused in the subsequent sintering process to form Sn–Co alloys composed of a large number of CoSn intermetallics and trace CoSn_2_ intermetallics. These Sn–Co alloys with grain diameters of about 5~15 nm are uniformly anchored on graphene. Increasing the sintering temperature can effectively improve the first Coulomb efficiency and cycle performance of the composites. The first charge capacity and Coulomb efficiency of Sn–Co alloy/rGO composites sintered at 450 °C are 675 mAh·g^−1^ and 80.4%, showing high first Coulomb efficiency. The continuous increase of sintering temperature will lead to a decrease in cycle performance, which may be caused by grain growth during the sintering process. The above results provide a strategy and technical approach for the synthesis of anode materials for lithium–ion batteries with high first Coulomb efficiency and good cycle performance.

## Figures and Tables

**Table 1:** Fitting circuit impedance parameters and lithium–ion diffusion coefficient of Sn–Co alloy/rGO composites.

Sintering Temperature	*R*_s_/Ω	*R*_SEI_/Ω	*R*_ct_/Ω	*D*_Li_^+^ × 10^−13^
Unsintered	4.84	38.67	46.32	2.16
400 °C	3.42	32.47	38.51	2.22
450 °C	2.35	28.24	31.05	2.96
500 °C	2.30	24.29	21.86	3.41
600 °C	2.32	24.43	25.93	3.07

## Data Availability

No new data were created or analyzed in this study. Data sharing is not applicable to this article.

## Supplementary Materials

The following supporting information can be downloaded at: https://www.mdpi.com/article/10.3390/molecules28093923/s1, Figure S1: The SEM of Sn–Co alloy/rGO composite with different sintering temperature Unsinted (a), 400 °C (b), 450 °C (c), 600 °C (d). Table S1: The long cycle test of Sn–Co alloy/rGO composite sintered at 450 °C. Table S2: Electrochemical performance of the reported Sn-based material for lithium–ion battery.
