# Supplementary material for: High Coulomb Efficiency Sn–Co Alloy/rGO Composite Anode Material for Li–ion Battery with Long Cycle–Life
Source: Molecules. 2023 May 6;28(9):3923. doi: 10.3390/molecules28093923 (PMC10179881; doi:10.3390/molecules28093923)
Supplement: Supplementary file 1 [file molecules-28-03923-s001.zip › molecules-2387987-SI.pdf]

High Coulomb efficiency Sn–Co alloy/rGO composite anode material for Li–ion battery with long cycle–life

Ding Shen [1], Mengyuan Jia [1], Mingyue Li [1,2], Xiaofan Fu [1], Yaohan Liu [1], Wei Dong [1,*] and Shaobin Yang [1,*]

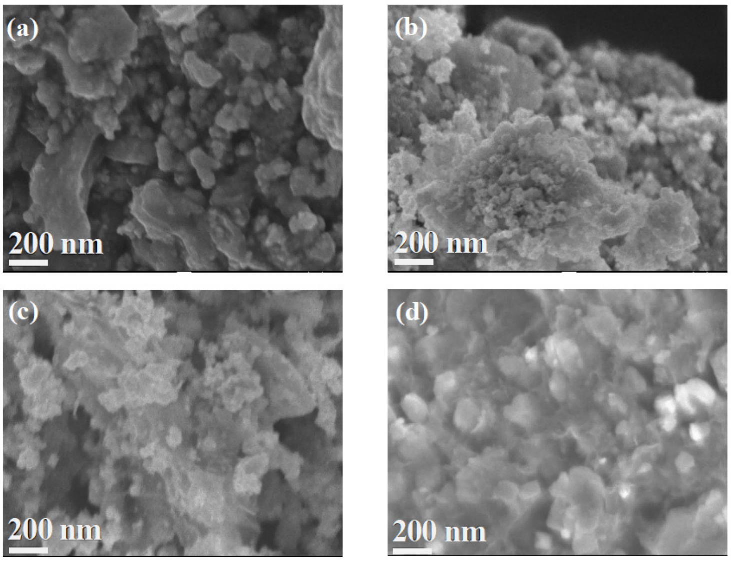

Figure S1. The SEM of Sn–Co alloy/rGO composite with different sintering temperature Unsinted (a), 400 °C (b), 450 °C (c), 600 °C (d)

Table S1. The long cycle test of Sn–Co alloy/rGO composite sintered at 450 °C

Button battery	The initial charge capacity / mAh·g^{-1}	The charge capacity after 500 cycles / mAh·g^{-1}	Capacity retention rate / %
1	628	445	70.9
2	622	443	71.2
3	615	446	72.5

Table S2. Electrochemical performance of the reported Sn–based material for lithium ion battery

Materials	Capacity	Cycle performance	First cycle Coulombic efficiency	Ref
Sn/DGT	913 mAh·g^{-1} at 0.2 A/g	100.5% after 500 cycles	71.1%	[16]
Sn–GNS	1407 mAh·g^{-1} at 0.08 A/g	63.9% after 30 cycles	65.9%	[17]
Sn@G–PGNWs	1245 mAh·g^{-1} at 0.2 A·g^{-1}	54.8% after 1000 cycles	69.1%	[19]
Sn@NG	1054 mAh·g^{-1} at 1 A·g^{-1}	53.9% after 1000 cycles	52.1%	[22]
Sn–Cu–GNS	525 mAh·g^{-1} at 0.5 A·g^{-1}	122.5% after 100 cycles	63.6%	[26]
SnCo/NC	1017 mAh·g^{-1} at 0.2 A/g	79.6% after 600 cycles	63.8%	[27]
$Fe_{0.74}Sn_5$@RGO	957 mAh·g^{-1} at 0.05 A/g	70.4% after 100 cycles	62.9%	[28]
Sn–Co/rGO	675 mAh·g^{-1} at 0.1 A/g	78.6% after 500 cycles	80.4%	**This work**
